# Building of CuO_2_@Cu-TA@DSF/DHA Nanoparticle Targets MAPK Pathway to Achieve Synergetic Chemotherapy and Chemodynamic for Pancreatic Cancer Cells

**DOI:** 10.3390/pharmaceutics16121614

**Published:** 2024-12-19

**Authors:** Jiaru Zhang, Zuoping Li, Zhenzhen Xie, Shiwan You, Yanbing Chen, Yuling Zhang, Jing Zhang, Na Zhao, Xiling Deng, Shiguo Sun

**Affiliations:** 1 Key Laboratory of Xinjiang Phytomedicine Resource and Utilization, Ministry of Education, College of Pharmacy, Shihezi University, Shihezi 832003, China; zhangjiaru@stu.shzu.edu.cn (J.Z.); lizuoping@stu.shzu.edu.cn (Z.L.); xiezz@stu.shzu.edu.cn (Z.X.); youshiwan@stu.shzu.edu.cn (S.Y.); 20192015023@stu.shzu.edu.cn (Y.C.); lynn723282347@stu.shzu.edu.cn (Y.Z.); zhangjing2@stu.shzu.edu.cn (J.Z.); zhaona@shzu.edu.cn (N.Z.); dxl_pha@shzu.edu.cn (X.D.); 2 Key Laboratory of Xinjiang Phytomedicine Resource and Utilization, Ministry of Education, College of Chemistry and Chemical Engineering, Shihezi University, Shihezi 832002, China; 3 Shanxi Key Laboratory of Natural Products & Chemical Biology, College of Chemistry & Pharmacy, Northwest Agriculture and Forestry University, Xianyang 712100, China; 4 Shenzhen Research Institute, Northwest Agriculture and Forestry University, Shenzhen 518000, China

**Keywords:** dihydroartemisinin, disulfiram, chemotherapy, chemodynamic, synergetic therapy

## Abstract

**Background/Objectives:** With the increase of reactive oxygen species (ROS) production, cancer cells can avoid cell death and damage by up-regulating antioxidant programs. Therefore, it will be more effective to induce cell death by using targeted strategies to further improve ROS levels and drugs that inhibit antioxidant programs. **Methods:** Considering that dihydroartemisinin (DHA) can cause oxidative damage to protein, DNA, or lipids by producing excessive ROS, while, disulfiram (DSF) can inhibit glutathione (GSH) levels and achieve the therapeutic effect by inhibiting antioxidant system and amplifying oxidative stress, they were co-loaded onto the copper peroxide nanoparticles (CuO_2_) coated with copper tannic acid (Cu-TA), to build a drug delivery system of CuO_2_@Cu-TA@DSF/DHA nanoparticles (CCTDD NPs). In response to the tumor microenvironment, DHA interacts with copper ion (Cu^2+^) to produce ROS, and a double (diethylthiocarbamate)-copper (II) (CuET) is generated by the complexation of DSF and Cu^2+^, which consumes GSH and inhibits antioxidant system. Meanwhile, utilizing the Fenton-like effect induced by the multi-copper mode can achieve ROS storm, activate the MAPK pathway, and achieve chemotherapy (CT) and chemodynamic (CDT). **Results:** Taking pancreatic cancer cell lines PANC-1 and BxPC-3 as the research objects, cell line experiments in vitro proved that CCTDD NPs exhibit efficient cytotoxicity on cancer cells. **Conclusions:** The CCTDD NPs show great potential in resisting pancreatic cancer cells and provides a simple strategy for designing powerful metal matrix composites.

## 1. Introduction

Pancreatic cancer is one of the common tumors in the digestive system, with a high degree of malignancy, and its treatment is still inadequate because of its unclear cause. At present, surgery, radiotherapy, chemotherapy, immunotherapy and sometimes their combinations are used to treat cancer [1,2]. However, the lack of early and effective diagnostic methods and means, low targeting, long-term side effects, systemic toxicity and multidrug resistance limit the effectiveness of these therapies [3,4,5,6,7,8,9,10,11]. Recently, chemical kinetic therapy (CDT) based on ROS storm strategy has received close attention in the field of tumor therapy. The purpose of ROS storm is to produce excessive ROS, and it is based on cell iron death [12]. It is reported that reactive oxygen species (ROS) plays an important role in regulating various cell processes, such as signal transduction, proliferation and survival, and promoting oxidative damage and cell death [13,14,15]. The balance between intracellular ROS and antioxidant programs is a crucial factor in maintaining cellular function in both normal and cancerous cells [16]. Its low concentration can stimulate the proliferation and invasion of cancer cells, while an excessive accumulation of ROS leads to oxidative stress, protein damage, and apoptosis [17]. Notably, cancer cells can counteract the injurious effects of ROS by up-regulating their antioxidant programs in response to increased ROS production. This adaptive mechanism enables cancer cells to evade cell death and injury. The augmentation of antioxidant activity neutralizes ROS and prolongs the survival of cancer cells [18]. Glutathione (GSH) represents the most abundant antioxidant in cells, playing a pivotal role in the neutralization of ROS. In this mechanism, the sulfhydryl group of cysteine provides a reducing equivalent to ROS [19]. By employing targeted strategies aiming to boost ROS levels while simultaneously administering drugs that inhibit antioxidant programs, cell death can be more effectively induced [20,21]. Dihydroartemisinin (DHA) has been studied as an innovative anticancer agent with significant cytotoxicity against various cancer cells in vitro and vivo [22,23,24]. It has been proven that DHA, due to its internal peroxide bridge containing sesquiterpene structures, can be cleaved by Fenton-like ions (such as Fe^2+^, Mn^2+^, and Cu^2+^), leading to the production of C-centered free radicals (•C) and enhancing intracellular oxidative stress [25,26]. The effect of DHA on cancer cells may be related to the production of ROS, which can cause oxidative damage to proteins, DNA, or lipids by producing excessive ROS, thereby inducing cancer cell apoptosis. Thus, DHA’s effectiveness against cancer cells may be attributed to its capacity to generate ROS and induce cancer cell apoptosis [27]. At this point, upon encountering high levels of reactive oxygen species (ROS), the antioxidant system is stimulated to neutralize these molecules. To augment the therapeutic impact on cancer cells, a compounded approach is taken, incorporating pharmaceuticals that inhibit both the antioxidant system and ROS generation [28]. This conjoined treatment protocol offers the added benefits of lower dosage requirements, mitigated side effects, and circumvention of drug resistance.

Furthermore, this approach may aid in suppressing or delaying metastasis [29]. Disulfiram (DSF) is an emerging and promising cancer chemotherapy drug, whose anticancer activity is formed by strong chelation with Cu ions (Cu^2+^) to form a double (diethylthiocarbamate)-copper (II) (CuET), which has a more significant anticancer activity against various cancers than the original DSF [30,31,32]. Zhang et al. [33] demonstrated that DSF can inhibit GSH levels and exert therapeutic effects by inhibiting the antioxidant system, amplifying oxidative stress [34]. NC Yip et al. [35] demonstrated that ROS plays a crucial role in regulating the MAPK pathway and cancer cell growth, inducing MAPK pathway activation. Activation of the MAPK pathway controls various cellular responses, including proliferation, differentiation, cell death, and survival [36,37,38]. In a bid to achieve enhanced treatment of cancer cells, DHA and DSF have been combined for the first time. The combination is expected to augment ROS levels, inhibit GSH levels, and regulate the MAPK pathway. This approach presents an opportunity to explore the potential of DHA and DSF in cancer treatment.

The interaction between Cu^2+^ and DHA has been shown to break the peroxide bridge and form a complex with DSF, resulting in a potent therapeutic effect [39,40]. To this end, copper peroxide nanoparticles (CuO_2_) were employed to co-deliver DHA and DSF, given that they possess a high drug-loading capacity and can release Cu^2+^ and H_2_O_2_ within cancer cells [41], This phenomenon is further augmented by the fact that Cu^2+^/Cu^+^ catalyze the production of ROS through Fenton-like reactions, which enhance the therapeutic efficacy of chemodynamic (CDT) treatment [42]. To further improve the poor stability of CuO_2_, tannic acid (TA), a phenolic hydroxyl compound [43,44], which can chelate with Cu^2+^ exhibiting strong coordination ability, is employed to deposit on the surface of CuO_2_, leading to the formation of CuO_2_@Cu-TA. In particular, the high affinity of Cu-TA to the CuO_2_ surface also contributes to the deposition of the Cu-TA network on CuO_2_, which can not only trigger and respond to the endogenous acidic pH of TME but also strengthens the stability of both the metal peroxide and the co-delivered DHA and DSF [45,46,47] and co-loaded DHA and DSF.

**Scheme 1:**
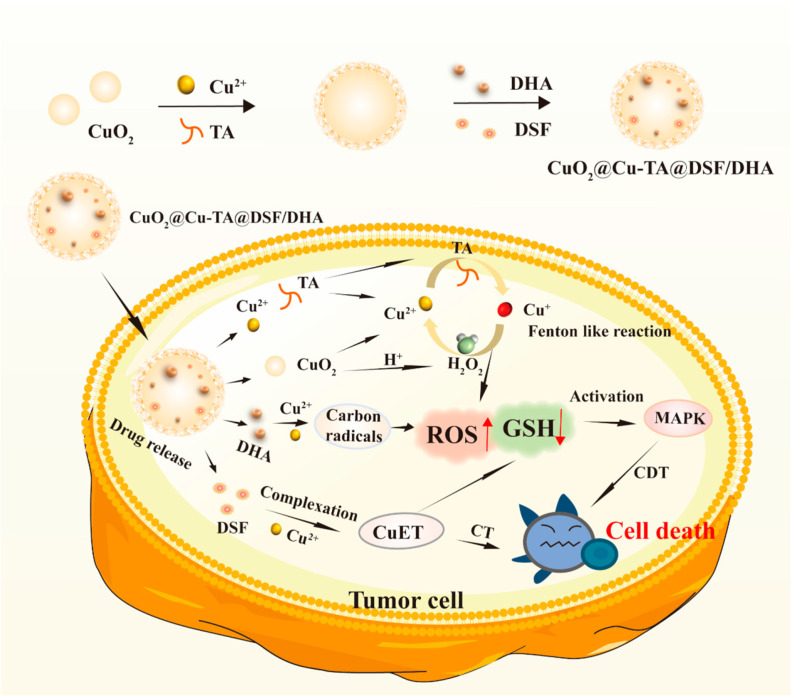
Building of CuO_2_@Cu-TA@DSF/DHA nanoparticle targets MAPK pathway to achieve synergetic CT and CDT for pancreatic cancer cells.

Studies have shown that for pancreatic cancer cells with a high ROS concentration, the increase of ROS level may increase the sensitivity of pancreatic cancer cell death, which is more influential than normal cells. Therefore, the process of elevating ROS has emerged as an efficacious treatment modality for pancreatic cancer cells [48]. In our work, a CuO_2_@Cu-TA@DSF/DHA nanoparticle drug loading system (CCTDD NPs) was constructed to achieve the Fenton-like effect induced by multi-copper mode, amplify the level of ROS, activate MAPK pathway, thereby consuming GSH and inhibiting the antioxidant system, and realize the synergistic treatment of chemotherapy (CT) and chemokinetic therapy (CDT). As shown in Scheme 1, firstly, CuO_2_ is prepared by a simple synthesis method and Cu-TA is deposited on its surface, and then DHA and DSF drugs are loaded by electrostatic adsorption. The cancer pancreatic cancer cell lines PANC-1 and BxPC-3 were selected as the research objects. Under the response of the tumor microenvironment, the drug delivery system cracks and releases Cu^2+^, DHA, and DSF. Cu^2+^ accelerates the rupture of DHA peroxide bridge and chelates with DSF to form CuET, and amplifies ROS level, thus realizing enhanced chemotherapy (CT). The reducibility of TA in an acidic environment promotes a Fenton-like reaction of Cu^2+^, further promotes the accumulation of ROS, achieves the CDT effect, and finally realizes synergetic CT/CDT therapy. In vitro ROS experiments and GSH experiments proved that CCTDD NPs can increase the level of ROS and consume GSH. In addition, CCTDD NPs can mediate the MAPK pathway to regulate pancreatic cancer cells and achieve effective treatment of pancreatic cancer cells.

## 2. Materials and Methods

### 2.1. Materials

Dihydroartemisinin (DHA) and Disulfiram (DSF) were purchased from MCE (MedChemExpress, Shanghai, China); TA was purchased from Macklin; phosphate buffer solution (PBS) was purchased from Shenggong Co. Ltd. (Shanghai, China); fetal bovine serum (FBS) was bought from Biological Industries Co. (shanghai, China); total GSH, Calcein-AM/PI, apoptosis and the reactive oxygen species assay kit were purchased from Beyotime (Shanghai, China); deionized water was supplied by a Milli-Q water system. All other chemicals were used without further purification.

### 2.2. Cell Lines

Human pancreatic cancer cell lines PANC-1 and BxPC-3 were obtained from the Institute of Biochemistry and Cell Biology at the Chinese Academy of Sciences, Shanghai, China. The cells were routinely cultured at 37 °C in DMEM and RPMI 1640 medium supplemented with 10% fetal calf serum, penicillin (100 U/mL), and streptomycin (100 mg/mL) in a CO_2_ incubator.

### 2.3. Cell Viability

The cell viability was tested using the MTT method, and cells were grown in 96-well plates for 24 h and then incubated with test samples [49]. Fresh medium containing 10% MTT was added to the cells, and they were incubated for 4 h. The formed formazan was dissolved using DMSO (Shanghai, China), and the absorption was measured at 490 nm using a microplate reader.

### 2.4. Live/Dead Staining Determination

PANC-1 and BxPC-3 cells were seeded in a 6-well plate at a density of 2 × 10^5^ per well for 24 h. Then, after 24 h of treatment with different dosing groups, all cells were collected by centrifugation and washed twice with PBS. Afterward, cells were stained with Calcein AM/PI (Shanghai, China) and detected through fluorescence imaging [50].

### 2.5. ROS and GSH Levels in Cells

The ROS and GSH levels in cells were measured using DCFH-DA and GSH assay kits [51]. This process involves treating cells with different administration groups of culture media and observing the results at different time points. After removing the culture medium, wash the cells were washed with PBS and incubated with the corresponding reagent kit for 20 min. The cells were then washed three times with an FBS-free culture medium and observed under a microscope.

### 2.6. Investigation of In Vitro Apoptosis

Apoptosis in different treatment groups. For the apoptosis assay, PANC-1 and BxPC-3 cells (2 × 10^5^ cells well^−1^) were treated with different treatment groups at the same concentration for 24 h. Then, the cells were washed twice with PBS, collected by centrifugation, and stained with Annexin V-FITC/PI (Shanghai, China) reagent kit to measure their apoptosis rate [52].

### 2.7. Synthesis of CuO_2_

CuO_2_ was prepared according to literature methods [53]. In short, 0.5 g of PVP was dissolved in 5 mL of CuCl_2_ (0.01 M) solution, to which 5 mL of NaOH (0.02 M) solution was added, and then stirred for some time. Next, 50 μL H_2_O_2_ (Wt: 30%) was added and stirred for half an hour. The final product was collected by ultrafiltration (8000 rpm/min, 4 min, 4 °C) and washed with water three times.

### 2.8. Preparation of CuO_2_@Cu-TA@DSF/DHA

Taking our optimized design as an example, 10 mg CuO_2_ was merged into a 10 mL centrifuge tube, and 5 mL CuCl_2_ (0.01 M) solution and 5 mL TA (0.01 M) solution were added to fully react. Subsequently, 5 mL of DSF (0.01 M) and 5 mL of DHA (0.01 M) solution were quickly mixed. Then the product was collected by high-speed centrifugation (8000 rpm/min, 4 min, 4 °C).

### 2.9. Characterization of CuO_2_@Cu-TA@DSF/DHA

The morphology and change of the Zeta potential of the nanoparticles were characterized by SEM (SEM; FEI Talos F200x, Waltham, MA, USA) and DLS (ZEN3600, Malvern, UK) measurement, respectively. Fourier transform infrared spectroscopy (FTIR) was recorded using an infrared spectrometer (Thermo Nicolet iS20, Waltham, MA, USA), and X-ray photoelectron spectroscopy was performed using an XPS spectrometer (Thermo ESCALAB 250Xi, Waltham, MA, USA), and X-ray diffraction spectra were measured using an X-ray diffractometer (Panalytical Empyrean, Almelo, The Netherlands).

### 2.10. Cellular Uptake CuO_2_@Cu-TA@DSF/DHA

Mark with Rhodamine B CuO_2_@Cu-TA@DSF/DHA was used to observe whether PANC-1 cells and B×PC-3 cells could uptake the nanocarrier system. Firstly, PANC-1 and BxPC-3 cells with logarithmic growth phase were inoculated into a six-well plate and incubated in a culture incubator for 24 h. Then, the cells were combined with those labeled with Rhodamine B CuO_2_@Cu-TA@DSF/DHA and the cells were treated with fresh medium for 2 h. Next, the cells were washed with PBS three times and stain the nuclei were stained with Hoechst 33342 for 5 min. Finally, the uptake of CuO_2_@Cu-TA@DSF/DHA by cells was observed using a fluorescence microscope.

### 2.11. Quantitative Real-Time Polymerase Chain Reaction (qPCR) Analysis

PANC-1 and BxPC-3 cells (2 × 10^5^ cell wells^−1^) were treated with different treatment groups at the same concentration for 24 h, and total RNA was extracted using Trizol reagent, followed by reverse transcription using a TransGen Biotech kit (TransGen Biotech, Beijing, China). Based on previous literature, qPCR was performed to analyze mRNA levels. The primer sequences are listed in Supplementary Materials: Table S1.

### 2.12. Western Blot Analysis

PANC-1 and BxPC-3 cells were inoculated in a 6-well plate with different treatment groups for 24 h. Subsequently, the cells were lysed, and collected, and the total protein concentration was measured using the BCA assay kit. Protein expression was measured using western blotting and Image J (1.4.3.67) software.

### 2.13. Statistical Analysis

Each experiment was repeated three times, and data were expressed as mean ± SD. Statistical comparisons of the results were performed using a student’s *t*-test. *p* < 0.05 is considered statistically significant.

## 3. Results and Discussion

### 3.1. Study on Anticancer of Drug Combination on PANC-1 Cells and BxPC-3 Cells

**Figure 1:**
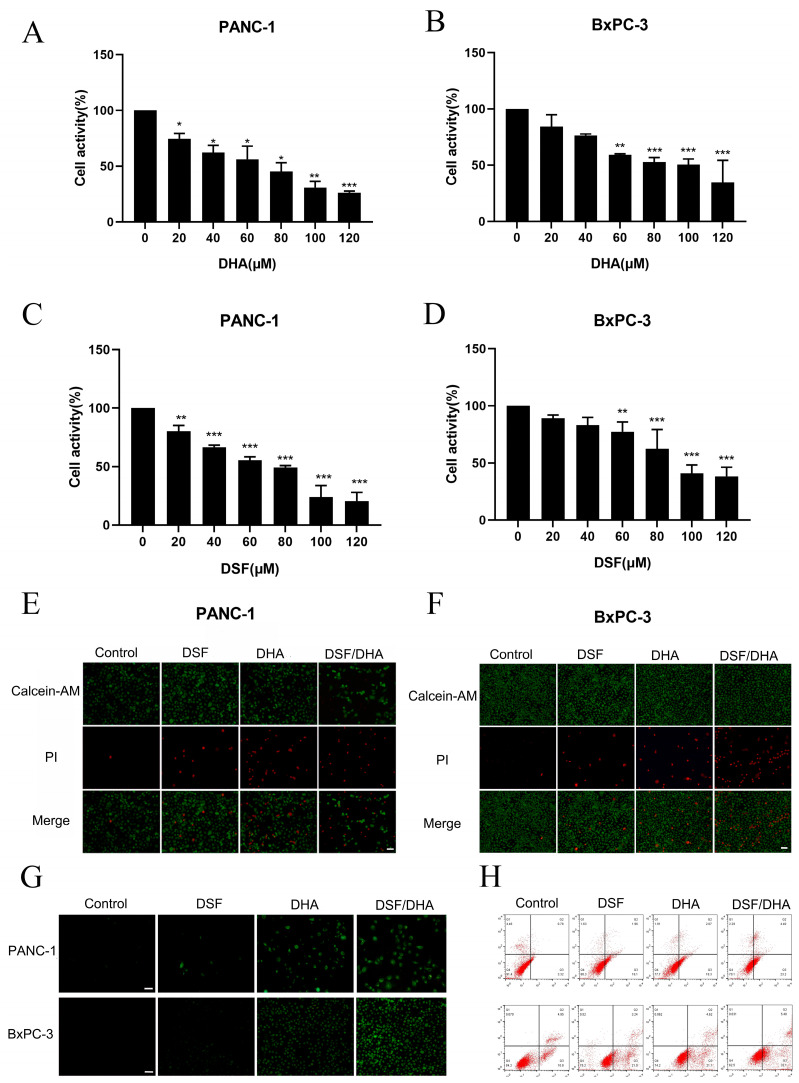
Inhibitory effect of drug combination on (**A**) PANC-1 cells and (**B**) BxPC-3 cells. Cell viability of (**C**) PANC-1 cells and (**D**) BxPC-3 cells treated with different concentrations of DHA. Evaluation of single and dual drugs by double staining of live/dead cells (Calcein-AM/PI) of (**E**) PANC-1 cells and (**F**) BxPC-3 cells (×10). (**G**) ROS generation levels after treatment with PANC-1 cells and BxPC-3 cells in different dosing groups (×10). (**H**) Apoptosis of PANC-1 cells and BxPC-3 cells treated with different administration groups. * *p* < 0.05, ** *p* < 0.01, *** *p* < 0.001.

To evaluate the combined effects of DHA and DSF on PANC-1 and BxPC-3, cytotoxicity was first evaluated. DHA and DSF inhibit the activity of PANC-1 cells and BxPC-3 cells in a concentration-dependent manner in the concentration range of 20 μM-120 μM (Figure 1A–D), which has a certain anti-pancreatic cancer effect. Secondly, this work continued to explore whether DHA and DSF have synergistic effects. We investigated the cell viability of PANC-1 and BxPC-3 cells in different ratios of DHA:DSF = 1:1, DHA:DSF = 1:2, and DHA:DSF = 2:1 through MTT experiments, as shown in Supplementary Materials (Figure S1). From the above figure and the investigation of the synergy index of Tables S1 and S2, it can be seen that when the ratio of DHA: DSF is 1:1, the synergy effect is the best. This ratio will be further investigated in the future. To further investigate the anti-cancer effect, the living and dead cells of PANC-1 and BxPC-3 cells were stained with Calcein-AM and PI kits, with red cells representing dead cells and green cells representing living cells. As shown in Figure 1E,F, compared with the control group, both the single drug group and the combined group have red fluorescence, and it is observed that the combined group has more obvious red fluorescence than the single drug group. Mitochondria is one of the biggest contributors of endogenous ROS, which can promote the apoptosis of tumor cells through DNA damage, lipid peroxidation, and protein damage, thus being closely related to the biological characteristics of tumors. Studies have shown that DHA can increase the level of ROS, and DSF can consume GSH. Therefore, to study whether DHA combined with DSF can synergistically amplify oxidative stress and increase the effect of ROS in pancreatic cancer cells PANC-1 and BxPC-3, DCFH-DA was used as a fluorescent probe detection index. As shown in Figure 1G, the effects on ROS in PANC-1 cells and BxPC-3 cells were investigated, respectively. The green fluorescence in the control group is the weakest and almost invisible; in contrast, the DSF group, DHA group, and DSF/DHA group showed green fluorescence, which confirmed the increase of ROS content in the cells. However, when the cells were exposed to the mixed culture medium of the DSF/DHA group, the green fluorescence was significantly higher than that of the control group and the single drug group. The results showed that the DSF/DHA group could synergistically increase the intracellular ROS content of pancreatic cancer cells PANC-1 and BxPC-3. ROS can promote tumor cell apoptosis through DNA damage, lipid peroxidation, and protein damage. Based on this, the apoptosis of pancreatic cancer cells PANC-1 and BxPC-3 induced by DHA and DSF was further discussed. Apoptosis was detected by AnnexinV-FITC/PI staining. As shown in Figure 1H, the percentage of early and late apoptotic cells increased after treatment in DSF, DHA, and DSF/DHA groups. The apoptosis rates of PANC-1 cells treated by the control group, DSF, DHA, and DSF/DHA groups were 4.1%, 18.06%, 20.07%, and 27.6%, respectively. The apoptosis rates of BxPC-3 cells treated by the control group, DSF, DHA, and DSF/DHA groups were 15.75%, 24.24%, 25.72%, and 37.5%, respectively. Compared with the control group, the early apoptosis rate of cells in the DSF and DHA groups increased, and the apoptosis of the DSF/DHA group was higher than that in the DSF and DHA groups. The results showed that the DSF/DHA group could enhance cancer cell apoptosis synergistically.

### 3.2. Preparation and Determination of CuO_2_@Cu-TA@DSF/DHA

**Figure 2:**
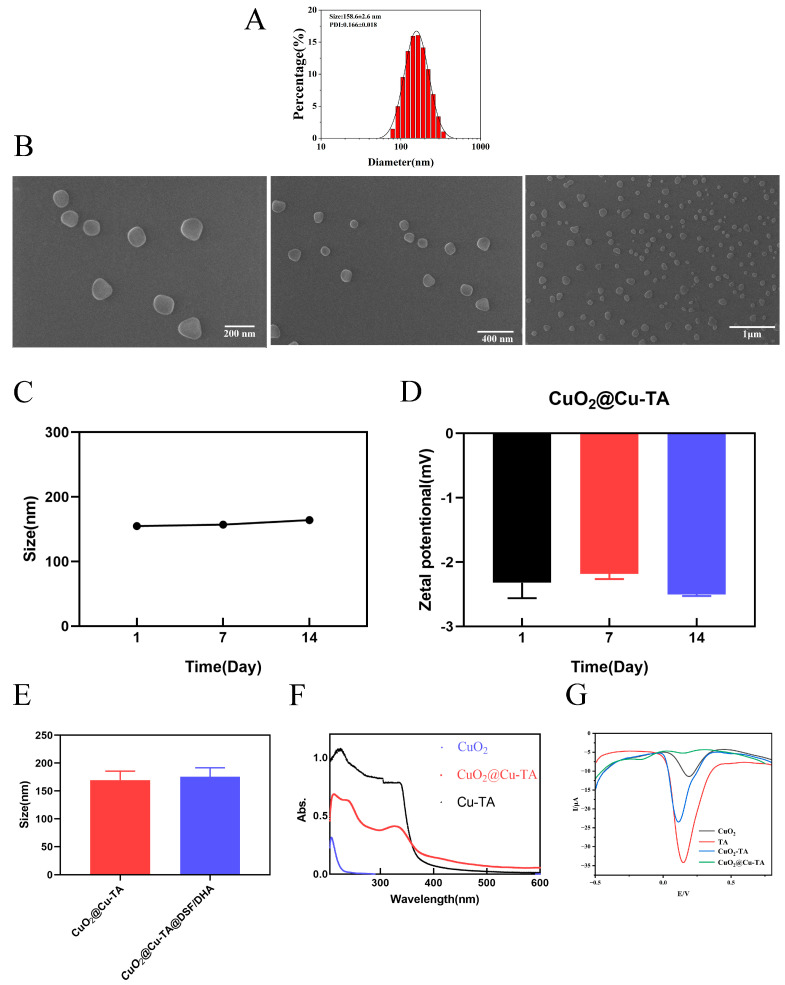
Preparation and determination of CuO_2_@Cu-TA@DSF/DHA. (**A**) Particle size distribution of CuO_2_@Cu-TA nanomaterials. (**B**) SEM characterization of CuO_2_@Cu-TA nanomaterials. (**C**) Particle size variation within 14 days. (**D**) Zeta variation within 14 days. (**E**) Particle size distribution with and without drug loading. (**F**) UV absorption spectrum of CuO_2_, Cu-TA, CuO_2_@Cu-TA. (**G**) Electrochemical test of CuO_2_, Cu-TA, CuO_2_@Cu-TA.

Co-delivery of drugs with nanocarriers has great advantages. The development of the copper peroxide-tannic acid dihydroartemisinin (CuO_2_@Cu-TA@DSF/DHA, CCTDD NPs) drug delivery system will be an effective way to transport DSF and DHA. In this paper, CuO_2_ was prepared using a simple method, and it was deposited on the surface of CuO_2_ through the coordination of Cu^2+^ and TA by electrostatic adsorption, thus forming a metal composite. As shown in Figure 2A, DLS test results show that CuO_2_@Cu-TA is about 158 nm. Studies have shown that nanoparticles have passive targeting ability when the particle size is less than 300 nm, which also proves that they can passively target cancer cells. In addition, in Figure 2B, the morphology of the particles was photographed by scanning electron microscope (SEM) at different magnifications, and it was found that CuO_2_@Cu-TA was spherical with uniform distribution, and CuO_2_@Cu-TA had good particle size and morphology, and this paper continues to study its stability. As shown in Figure 2C, the stability of CuO_2_@Cu-TA was observed by DLS within 14 days, and no obvious change was observed. In addition, as shown in Figure 2D, DLS also showed that the Zeta potential did not change significantly within 14 days, and the Zeta potential was negative, which would better enter the cells. Figure 2E shows the particle size change of nanoparticles after drug loading, and the particle size will increase slightly compared with before. To sum up, nanoparticles and drug-loaded nanoparticles have good stability and dispersibility, which lays a foundation for the subsequent study of drug delivery systems. To prepare CuO_2_@Cu-TA successfully, the whole wavelength was scanned by an ultraviolet-visible spectrophotometer, and its absorption spectrum and peak value were recorded. As shown in Figure 2F, CuO_2_ has an absorption peak, and CuO_2_@Cu-TA retains the characteristic absorption spectra of CuO_2_ and Cu-TA, which proves that CuO_2_@Cu-TA is tested electrochemically according to the redox reaction between molecules, and a solution with a certain concentration of CuO_2_, Cu-TA, and CuO_2_@Cu-TA is obtained. The unique electrochemical activity of each substance is distinguished according to its redox properties. As shown in Figure 2G, CuO_2_ has an obvious signal peak at about 0.2 V, and TA has an obvious signal peak at about 0.15 V. CuO_2_-TA composite has two signal peaks with similar positions because the peak positions of CuO_2_ and TA are close, while CuO_2_@Cu-TA composite has different signal peaks at three potentials, and its electronegativity has a certain shift. Based on this, the determination of CuO_2_@Cu-TA was successfully prepared.

### 3.3. Characterization of CuO_2_@Cu-TA@DSF/DHA

**Figure 3:**
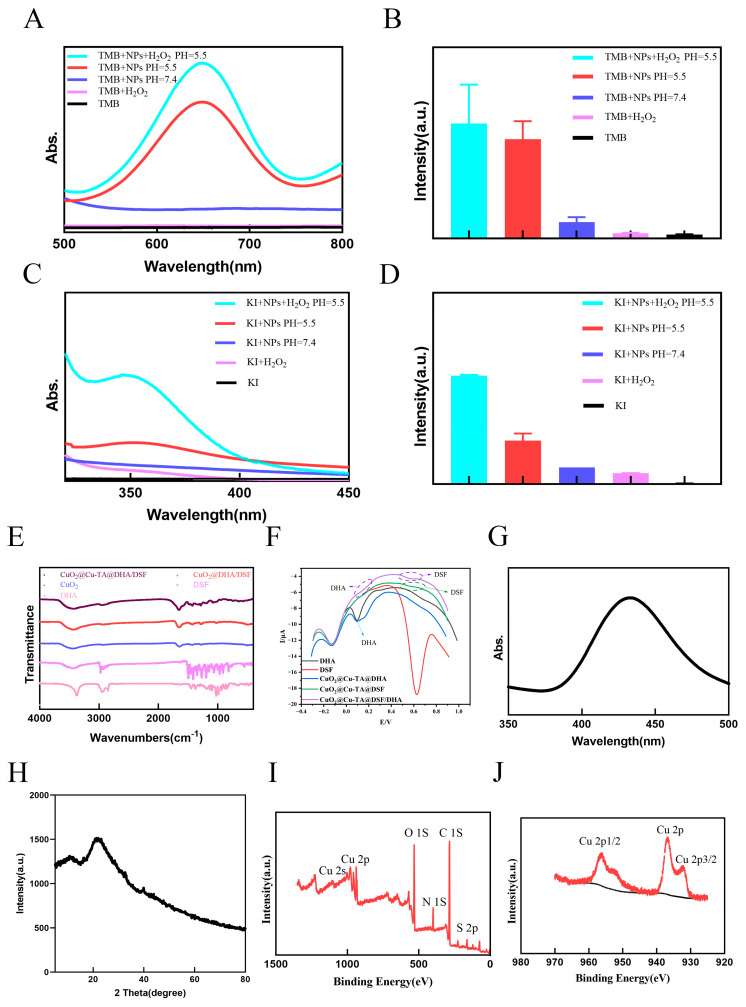
Characterization of CuO_2_@Cu-TA@DSF/DHA. (**A**) UV–vis spectra at about 650 nm. (**B**) Absorption bar chart. (**C**) UV–vis spectra at 350 nm. (**D**) Absorption bar chart. (**E**) FTIR spectra of each sample. (**F**) Electrochemical test of DHA, DSF, CuO_2_@Cu-TA@DSF, CuO_2_@Cu-TA@DHA, CuO_2_@Cu-TA@DSF/DHA. (**G**) UV spectrum of Cu^2+^ complexation with DSF. (**H**) XRD patterns of CuO_2_@Cu-TA@DSF/DHA. (**I**) XPS spectrum of CuO_2_@Cu-TA@DSF/DHA. (**J**) Cu 2p XPS spectrum of CuO_2_@Cu-TA@DSF/DHA.

To investigate the level of H_2_O_2_ produced by nanoparticles, TMB was used as an index to confirm the production of •OH in a Fenton-like reaction. The generation of •OH will oxidize colorless TMB into blue-green oxidized TMB, showing the maximum absorption at 652 nm. The UV-visible spectra of various reaction systems are shown in Figure 3A, and the histogram at about 650 nm is shown in Figure 3B. TMB and TMB H_2_O_2_ groups served as negative controls. When exposed to an acidic environment with a pH of 5.5, the color of the reaction system turned dark green; however, no obvious color change was observed in a neutral environment with a pH of 7.4. The experimental results show that the nanoparticles can mediate a Fenton-like reaction and produce •OH in an acidic environment without adding extra H_2_O_2_. In addition, when adding extra H_2_O_2_, they can mediate a stronger Fenton-like reaction and produce more •OH. To detect ROS, the oxidation reaction of KI was also carried out. As shown in Figure 3C,D, I^−^ can be oxidized to I^3−^ by ROS, and ROS has a maximum absorption peak at about 350 nm. The results obtained from the KI experiment show a similar trend to the TMB experiment. As shown in Figure 3E, the infrared spectra of each sample were evaluated by Fourier transform infrared (FTIR) spectra. At 553 cm^−1^, 1375 cm^−1^ and 1496 cm^−1^, the characteristic absorption of DSF was attributed to the tensile vibration of S-S, C=S, and C-N-C, respectively. The tensile vibration absorption of C=O (1648 cm^−1^) and O–H (3640–3160 cm^−1^) are the characteristic absorption peaks of DHA and TA. The peak of 1600–1400 cm^−1^ is attributed to benzene skeleton vibration, and the peak near 1200 cm^−1^ is attributed to CO tensile vibration. CuO_2_ only shows the characteristic peaks of PVP (1643, 1422, and 1287 cm^−1^) because it does not involve other organic materials. The above characteristic absorption peaks at CuO_2_@Cu-TA@DSF/DHA verify the coexistence of DSF, DHA, CuO_2_, and Cu-TA, proving that DSF and DHA are successfully loaded. In addition, according to the redox reaction between molecules of substances, the electrochemical test is carried out by the electrochemical method, and the unique electrochemical activity of each substance is distinguished by the difference in redox properties, as shown in Figure 3F. DHA, and DSF have obvious signal peaks at about 0.1 V and 0.6 V, respectively, and the signal peaks at 0.1 V and 0.6 V can be observed in the compound loaded with DHA or DSF alone. However, the complex loaded with DHA and DSF can observe signal peaks at 0.1 V and 0.6 V at the same time, which proves that DHA and DSF are successfully loaded in CuO_2_@Cu-TA. In addition, according to the ultraviolet characteristic spectra of DSF, the standard curve established to calculate the drug loading rate of DSF was 12.79%, respectively. Similarly, DHA were 10.98%. At the same time, to prove that DSF and Cu^2+^ will combine, the research shows that the ultraviolet absorption will have a characteristic absorption peak at 400–450 nm, as shown in Figure 3G, and the characteristic spectra will appear at 400–450 nm in an acidic environment. To sum up, the successful load of DSF and DHA is confirmed, which will provide a basis for subsequent research. As shown in Figure 3H, XRD results show that there are two peaks at 15° and 21°, which are similar to those reported in the literature and proved to be crystalline. As shown in Figure 3I, the XPS test shows that CuO_2_@Cu-TA@DSF/DHA is composed of Cu, C, N, O, and S, and the peak of 531 eV belongs to C=O, and the peak of 532 eV belongs to O-O in CuO_2_, which is very important for the self-supply of H_2_O_2_. As shown in Figure 3J, the spectrum of Cu 2p shows two prominent peaks at 932 eV and 953 eV, and satellite peaks at 938 eV and 957 eV, indicating the existence of Cu(II) form. Therefore, the above experiments proved the successful synthesis of CuO_2_@Cu-TA@DSF/DHA.

### 3.4. Synergistic Anticancer Effect of CuO_2_@Cu-TA@DSF/DHA In Vitro

**Figure 4:**
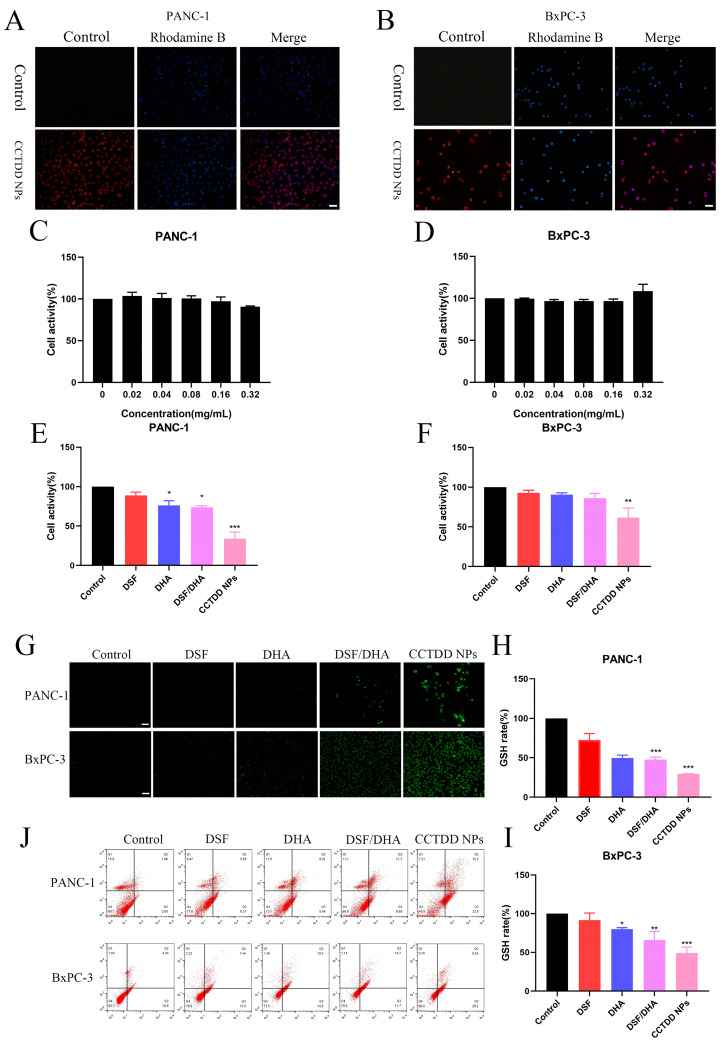
Synergistic anticancer effect of CuO_2_@Cu-TA@DSF/DHA in vitro. Fluorescence image of (**A**) PANC-1 cells and (**B**) BxPC-3 cells incubated with Rhodamine B-CCTDD NPs for 2 h and co-located with nuclear dye Hoechst33342 (×10). Different concentration treatment of CuO_2_@Cu-TA on cell viability of (**C**) PANC-1 cells and (**D**) BxPC-3 cells. The effect of different administration groups on the viability of (**E**) PANC-1 cells and (**F**) BxPC-3 cells. (**G**) Measurement of ROS fluorescence of PANC-1 cells and BxPC-3 cells after treatment with different administration groups (×10). GSH levels in (**H**) PANC-1 cells and (**I**) BxPC-3 cells after treatment in different treatment groups. (**J**) The effect of different treatment groups on the apoptosis rate of PANC-1 cells and BxPC-3 cells. * *p* < 0.05, ** *p* < 0.01, *** *p* < 0.001.

To investigate the uptake capacity of CuO_2_@Cu-TA@DSF/DHA (CCTDD NPs), Rhodamine B was used to label CCTDD NPs. As shown in Figure 4A,B, the uptake of PANC-1 cells and BxPC-3 cells were investigated respectively. In the control group, there was only a blue fluorescence signal, while in Rhodamine B labeled CCTDD NPs, there were strong red fluorescence signals in the cytoplasm of PANC-1 cells and BxPC-3 cells. The results showed that CCTDD NPs could be successfully endocytosed into cancer cells. Nano-material is a good drug delivery system, and its compatibility and safety cannot be ignored. As shown in Figure 4C,D, cells incubated with different concentrations of the blank carrier for 24 h still showed a high survival rate, and even incubated for 24 h at the concentration of 0.32 mg/mL, the cell survival rate still had no significant effect. It is proved that CuO_2_@Cu-TA has certain security. As shown in Figure 4E,F, the cell viability of different administration groups was investigated. From the figure, it can be seen that the CCTDD NPs group has significant cytotoxicity. To sum up, this drug delivery system has a good anti-pancreatic cancer effect. In the early stage, it was confirmed that DSF/DHA can enhance ROS. Here, we will explore the influence of CCTDD NPs on ROS level. Under the response of the tumor microenvironment, TA can transform Cu^2+^ into Cu^+^, and H_2_O_2_ into •OH, which can induce CDT to kill cancer cells through the Fenton reaction. However, CuO_2_ releases copper ions and H_2_O_2_ in the tumor environment, realizing enhanced CDT effect and producing obvious ROS. Therefore, the effects of different administration groups on intracellular ROS were evaluated. As shown in Figure 4G, the control group, DSF group, and DHA group, have weak fluorescence, the DSF/DHA group has certain fluorescence, and the CCTDD NPs group has the highest fluorescence. Therefore, it is confirmed that the large production of ROS in the CCTDD NPs group may be related to the synergistic effect between drugs and carriers. Increasing ROS level is accompanied by consumption of GSH levels. To detect the GSH level, as shown in Figure 4H,I, the GSH levels in PANC-1 cells and BxPC-3 cells were investigated respectively. As can be seen from the figure, compared with the control group, the DSF, DHA, and DSF/DHA groups have a certain GSH consumption capacity, while the CCTDD NPs group has a more obvious GSH consumption capacity than other groups. To sum up, the CCTDD NPs have good GSH consumption capacity. As shown in Figure 4J, the apoptosis levels of PANC-1 cells and BxPC-3 cells in different administration groups were investigated respectively. The apoptosis rates of PANC-1 cells treated by the control group, DSF group, DHA group, and DSF/DHA group were 3.91%, 14.16%, 14.39%, 20.35%, and 47.2%, respectively. The apoptosis rates of BxPC-3 cells treated by the control group, DSF group, DHA group, and DSF/DHA group were 15.29%, 20.94%, 25.1%, 28.4%, and 38.82%, respectively. Compared with other drug groups, the CCTDD NPs group showed the highest apoptosis rate. The CCTDD NPs have a good anti-pancreatic cancer effect.

### 3.5. Study on Anticancer Mechanism In Vitro

**Figure 5:**
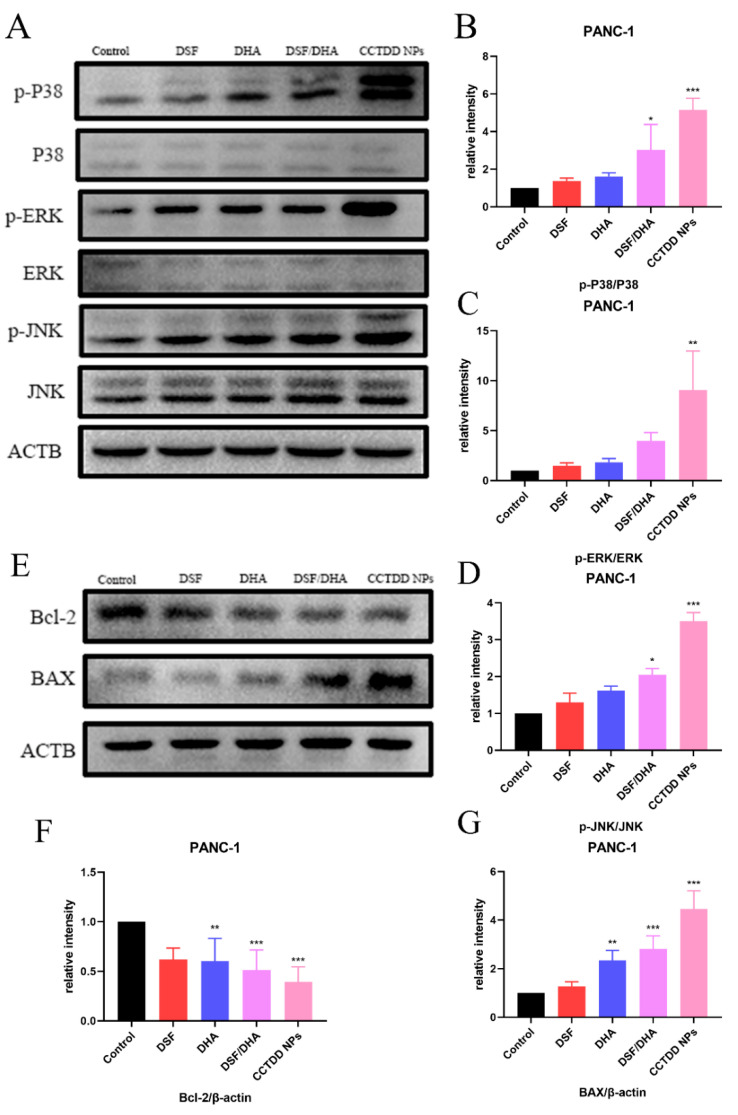
Study on anticancer mechanism in vitro. (**A**) Western blot analysis of P38, ERK, and JNK protein expression levels in PANC-1 cells treated with different administration groups. (**B**) Quantitative analysis of p-P38/P38. (**C**) Quantitative analysis of p-ERK/ERK. (**D**) Quantitative analysis of p-JNK/JNK. (**E**) Western blot analysis of Bcl-2 and Bax protein expression levels in PANC-1 cells treated with different administration groups. (**F**) Quantitative analysis of Bcl-2. (**G**) Quantitative analysis of Bax. * *p* < 0.05, ** *p* < 0.01, *** *p* < 0.001.

A large number of studies show that the production of ROS will activate MAPK to play a role, and different MAPK pathways will play different biological roles. The proliferation and differentiation of cells are realized through the ERK signaling pathway; apoptosis and inflammatory reaction of cells are realized through JNK and P38. Therefore, we made a preliminary study through a qPCR experiment. As shown in Supplementary Figure S2, compared with other groups, the mRNA levels of JNK, ERK, and P38 of the CCTDD NPs group were significantly increased, which preliminarily proved that CCTDD NPs may play a role through MAPK. Figure 5A is the western blot analysis chart of MAPK pathway-related proteins, and Figure 5B–D is the quantitative analysis chart. Compared with other groups, the protein expression levels of JNK, ERK, and P38 in the CCTDD NPs group have not changed, but the protein expression levels of p-JNK, p-ERK, and p-P38 have increased significantly. Figure 5E is the protein western blot analysis chart of Bcl-2 and Bax, and Figure 5F,G is the quantitative analysis chart. After the CCTDD NPs group treatment, the expression of Bcl-2 protein is the least, and the expression of Bax protein is the most. It can be proved that CCTDD NPs may mediate the biological process of cells through the MAPK signaling pathway.

## 4. Conclusions

In summary, a biodegradable nano drug delivery system CuO_2_@Cu-TA@DSF/DHA has been developed (CCTDD NPs) and is used to deliver DSF and DHA to enhance combination therapy. In vitro experiments have shown that CuO_2_@Cu-TA @DSF/DHA can release Cu^2+^-mediated Fenton-like reactions in tumor microenvironment and produce •OH without the need for additional H_2_O_2_. It is interesting that when additional H_2_O_2_ is added, CCTDD NPs can mediate stronger Fenton-like reactions and produce more •OH. The results of cytotoxicity test and flow cytometry showed that CCTDD NPs had a good anti-pancreatic cancer effect. This is mainly due to the interaction between Cu^2+^ and DHA, which enhances the drug’s enhancement effect and achieves ROS amplification through the cleavage of internal peroxide bridges mediated by Cu^2+^ and the formation of toxic free radicals. At the same time, Cu^2+^ chelates with DSF to form CuET, consumes GSH, enhances ROS, and enhances the efficacy of DSF. In addition, western blot results showed that CCTDD NPs significantly increased the protein expression of p-JNK, p-ERK, and p-P38, while the protein expression of JNK, ERK, and P38 was basically unchanged, indicating that CCTDD NPs can regulate the MAPK pathway to achieve the effect of treating pancreatic cancer cells. The therapeutic effect of this therapeutic nano platform has been confirmed through in vitro experiments, in which the growth of pancreatic cancer cells is significantly inhibited, and its toxic effect on healthy tissues/cells can be ignored. In conclusion, this nano drug loading system can play a role by enhancing ROS production and regulating MAPK pathway, which will provide a basis for pancreatic cancer synergistic treatment and provide new strategies for pancreatic cancer treatment.

## Data Availability

Data will be made available on request.

## Supplementary Materials

The following supporting information can be downloaded at: https://www.mdpi.com/article/10.3390/pharmaceutics16121614/s1, Figure S1: The effect of different drug ratios on cell viability; Table S1: IC50 examination under single drug and different combination ratios; Table S2: IC50 examination under single drug and different combination ratios; Figure S2: mRNA levels in PANC-1 cells and BxPC-3 cells treated with different administration groups.
