# Supplementary material for: Building of CuO2@Cu-TA@DSF/DHA Nanoparticle Targets MAPK Pathway to Achieve Synergetic Chemotherapy and Chemodynamic for Pancreatic Cancer Cells
Source: Pharmaceutics. 2024 Dec 19;16(12):1614. doi: 10.3390/pharmaceutics16121614 (PMC11680075; doi:10.3390/pharmaceutics16121614)
Supplement: Supplementary file 1 [file pharmaceutics-16-01614-s001.zip › pharmaceutics-3348051-supplementary.pdf]

Supporting Information

Building of CuO_2@Cu-TA@DSF/DHA Nanoparticle Targets MAPK Pathway to Achieve Synergetic Chemotherapy and Chemodynamic for Pancreatic Cancer Cells

Jiaru Zhang [1,†], Zuoping Li [1,2,†], Zhenzhen Xie [1,2], Shiwan You [1], Yanbing Chen [1,2], Yuling Zhang [1,2], Jing Zhang [1,2], Na Zhao [1], Xiling Deng [1] and Shiguo Sun [1,3,4,*]

[1] Key Laboratory of Xinjiang Phytomedicine Resource and Utilization,Ministry of Education,
College of Pharmacy, Shihezi University, Shihezi 832003, China;
zhangjiaru@stu.shzu.edu.cn (J.Z.); lizuoping@stu.shzu.edu.cn (Z.L.);
xiezz@stu.shzu.edu.cn (Z.X.); youshiwan@stu.shzu.edu.cn (S.Y.);
20192015023@stu.shzu.edu.cn (Y.C.); lynn723282347@stu.shzu.edu.cn (Y.Z.);
zhangjing2@stu.shzu.edu.cn (J.Z.); zhaona@shzu.edu.cn (N.Z.);
dxl_pha@shzu.edu.cn (X.D.)

[2] Key Laboratory of Xinjiang Phytomedicine Resource and Utilization, Ministry of Education,
College of Chemistry and Chemical Engineering, Shihezi University, Shihezi 832002, China

[3] Shanxi Key Laboratory of Natural Products & Chemical Biology, College of Chemistry & Pharmacy, Northwest Agriculture and Forestry University, Xianyang, 712100, China

[4] Shenzhen Research Institute, Northwest Agriculture and Forestry University, Shenzhen 518000, China

* Correspondence: sunsg@nwsuaf.edu.cn; Tel.: +86-13898652147

† These authors contributed equally to this work.

Academic Editors: Murali Mohan Yallapu and Nebojša Pavlović

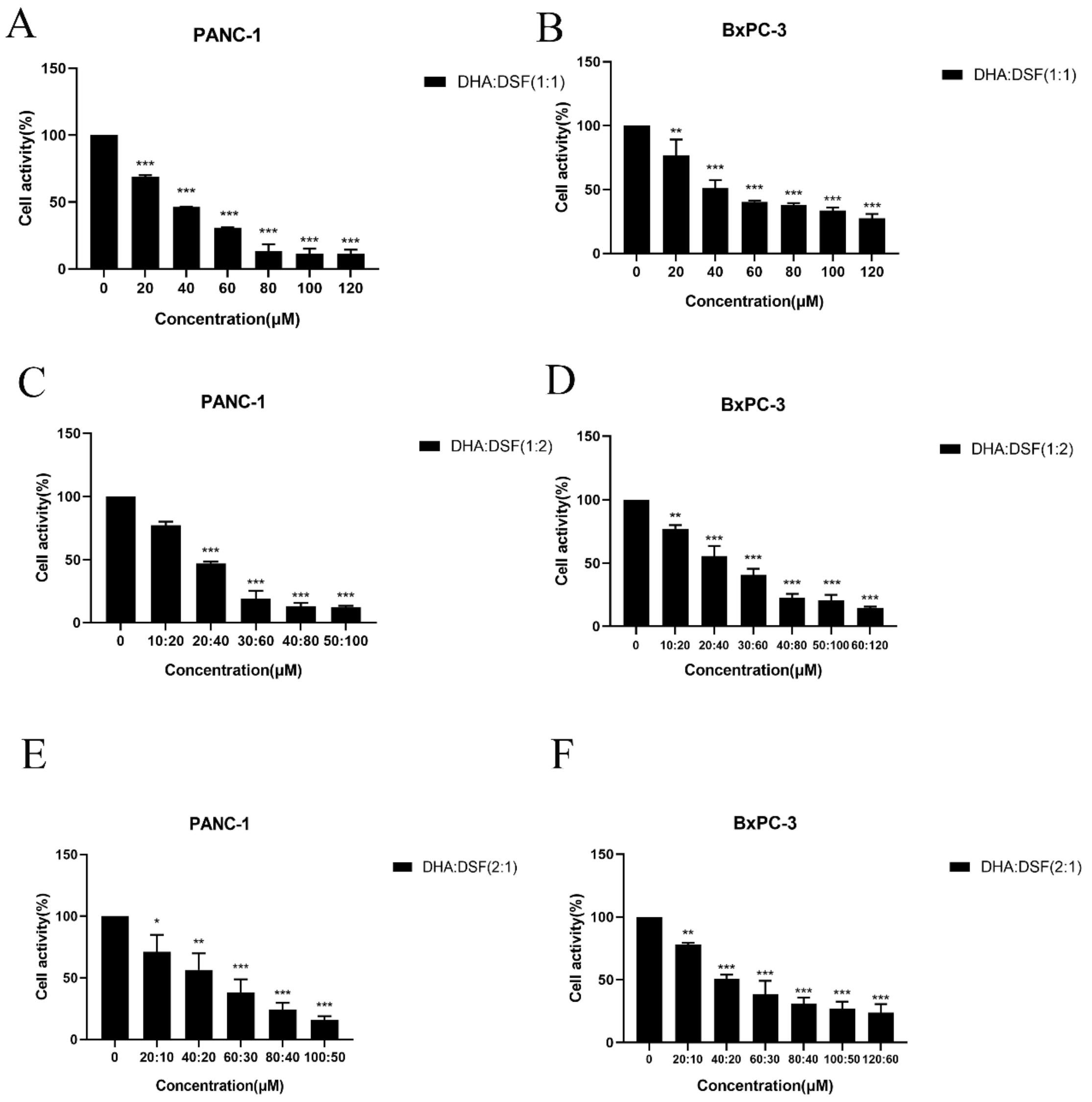

Figure S1. The effect of different drug ratios on cell viability. (A) The effect of DHA：DSF = 1：1 on the viability of PANC-1 cells. (B) The effect of DHA：DSF = 1：1 on the viability of BxPC-3 cells. (C) The effect of DHA：DSF = 1：2 on the viability of PANC-1 cells. (D) The effect of DHA：DSF = 1：2 on the viability of BxPC-3 cells. (E) The effect of DHA：DSF = 2：1 on the viability of PANC-1 cells. (F) The effect of DHA：DSF = 2：1 on the viability of BxPC-3 cells

Table S1. IC_{50} examination under single drug and different combination ratios

PANC-1	DHA(IC_{50})	DSF(IC_{50})	Con(IC_{50})	BxPC-3	DHA(IC_{50})	DSF(IC_{50})	Con(IC_{50})
	61.47				86.16		
		61.86				94.45	
1：1			34.07	1：1			48.29

1：2			53.48	1：2			66.04
2：1			61.95	2：1			67.41

Table S2. IC_{50} examination under single drug and different combination ratios

PANC-1	DHA(IC_{50})	DSF(IC_{50})	Con(IC_{50})	BxPC-3	DHA(IC_{50})	DSF(IC_{50})	Con(IC_{50})
	61.47				86.16		
		61.86				94.45	
1：1			34.07	1：1			48.29
1：2			53.48	1：2			66.04
2：1			61.95	2：1			67.41

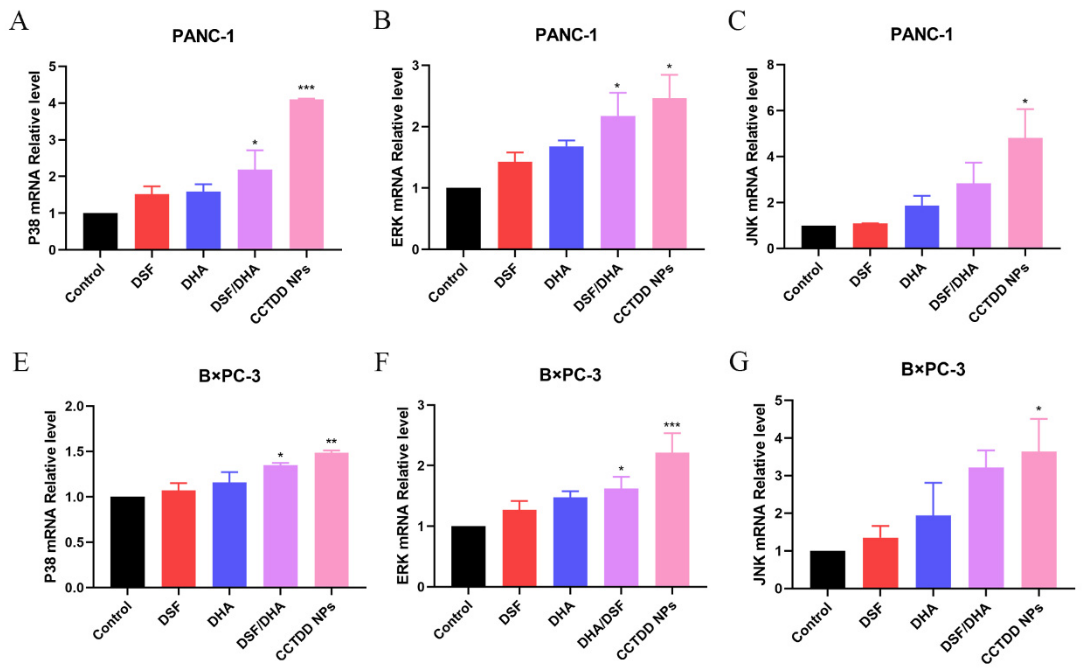

Figure S2. mRNA levels in PANC-1 cells and BxPC-3 cells treated with different administration groups. A. B and C represent the mRNA levels of P38, ERK, and JNK in PANC-1 cells, respectively; D. E and F represent the mRNA levels of P38, ERK, and JNK in BxPC-3 cells, respectively
